# Prognosis and modulation mechanisms of COMMD6 in human tumours based on expression profiling and comprehensive bioinformatics analysis

**DOI:** 10.1038/s41416-019-0571-x

**Published:** 2019-09-16

**Authors:** Mi Yang, Weiqiang Huang, Yaling Sun, Huazhen Liang, Min Chen, Xixi Wu, Xiaoqing Wang, Longshan Zhang, Xiaoya Cheng, Yao Fan, Hua Pan, Longhua Chen, Jian Guan

**Affiliations:** 1 Department of Radiation Oncology, Nanfang Hospital, Southern Medical University, Guangdong, Guangzhou China; 2 Department of Radiation Oncology, The Third Affiliated Hospital, Sun Yat-sen University, Guangdong, Guangzhou China

**Keywords:** Tumour biomarkers, Bioinformatics

## Abstract

**Background:**

The Copper Metabolism MURR1 (COMM) domain family has been reported to play important roles in tumorigenesis. As a prototype for the COMMD family, the expression pattern and biological function of COMMD6 in human tumours remain unknown.

**Methods:**

COMMD6 expression in BALB/c mice and human tissues was examined using real-time PCR and immunohistochemistry. Kaplan–Meier analysis was applied to evaluate the prognosis of COMMD6 in tumours. Competing endogenous RNA (ceRNA) and transcriptional regulation network were constructed based on differentially expressed mRNAs, microRNAs and long non-coding RNAs from the cancer genome atlas database. GO and KEGG enrichment analysis were used to explore the bioinformatics implication.

**Results:**

COMMD6 expression was widely observed in BALB/c mice and human tissues, which predicted prognosis of cancer patients. Furthermore, we shed light on the underlying tumour promoting role and mechanism of COMMD6 by constructing a TEX41-miR-340-COMMD6 ceRNA network in head and neck squamous cell carcinoma and miR-218-CDX1-COMMD6 transcriptional network in cholangiocarcinoma. In addition, COMMD6 may modulate the ubiquitination and degradation of NF-κB subunits and regulate ribonucleoprotein and spliceosome complex biogenesis in tumours.

**Conclusions:**

This study may help to elucidate the functions and mechanisms of COMMD6 in human tumours, providing a potential biomarker for tumour prevention and therapy.

## Background

The incidence and mortality of cancer are rapidly growing worldwide. The global cancer burden is further aggravated with an estimated 18.1 million new cancer cases and 9.6 million cancer deaths in 2018, being the leading cause of death. The cumulative risks of developing or dying from cancer before age 75 years globally are 21.4 and 17.7%, respectively.^1^ Thus, the prevention and treatment of cancer has become an urgent global challenge in nowadays.

The occurrence and development of malignant tumours is a multi-step and multi-stage complex process.^2^ The hallmarks of cancer development include sustaining proliferative signalling, evading growth suppressors, resisting cell death, enabling replicative immortality, inducing angiogenesis and activating invasion and metastasis.^3^ Gene dysregulation, such as activation of oncogenes and inactivation of tumour suppressing genes, is a common trait of human cancers and have been involved in each of these hallmarks.^4,5^ Therefore, a better understanding of key tumour-driven genes will help to elucidate the mechanism of tumorigenesis at the molecular level, as well as improve the accurate prevention and treatment of malignant tumours.

The COMM domain family, which contains 10 family members all sharing a highly conserved domain in their extreme carboxy-terminus,^6^ has been reported to play important roles in tumorigenesis. As the best characterised member of the COMMD family, COMMD1 induces apoptosis in human lung cancer cells by inhibition of SOD1.^7^ Increased nuclear expression of COMMD1 suppresses cyclinD1 expression, cell proliferation of neuroblastoma cells.^8^ Moreover, overexpression of COMMD1 inhibits colorectal cancer, glioblastoma and melanoma cells invasion and metastasis by directly targeting of HIF.^9^ It is also found that nuclear expression of COMMD1 sensitises ovarian cancer to cisplatin,^10^ and downregulation COMMD1 promotes tumour development by modulating a positive feedback loop that amplifies inflammatory- and stemness-associated properties of cancer cells.^11^ In addition, COMMD5 inhibits renal cell carcinoma by promoting de-phosphorylation of ErbB2/HER2 and epigenetic gene silencing of EGFR and ErbB3 via promoter methylation. COMMD7 has been found to promote cell migration and invasion of hepatocellular carcinoma by upregulating PIAS4^12^ or CXCL10.^13^ It has also been found that inhibition of COMMD7 suppresses invasion of pancreatic ductal adenocarcinoma by decreasing MMP2 secretion.^14^ COMMD9 promotes TFDP1/E2F1 activation and progression of non-small cell lung cancer by interaction with TFDP1.^15^ Moreover, we have previously shown the expression profiles of COMMD10 in human tumours^16^ and found that COMMD10 inhibits the invasion and metastasis of colorectal cancer by promoting ubiquitination and degradation of NF-kB.^17^

As a prototype for the COMMD family, COMMD6 was primarily formed by the COMM domain, a region of the COMMD proteins that is crucial for multiple biological functions.^18^ COMMD6 from amphioxus Branchiostoma belcheri forms a heterodimer with creatine kinase, leading to inhibition of creatine kinase activity and energy conversion.^19^ COMMD6 colocalises with the WASH complex and retromer in sub-compartment of the endosome and participates in the CCC-WASH axis in endosomal sorting of receptors.^20^ Moreover, COMMD6 has also been reported to be involved in the inhibition of NF-κB pathway activity in HEK-293 cells. The activation of NF-κB could be completely abolished by the mutation of the amino acid residues Trp24 and Pro41 in the COMM domain of COMMD6.^21^ Aberrant activation of the NF-κB signalling pathway has been observed in many human cancers, which induce tumour infinite growth and progression.^22^ Thus, we assumed that COMMD6 may be involved in the tumorigenesis and malignant progression. However, the tissue-specific expression, biology function and the possible mechanism of COMMD6 in tumour remain unknown. Here, we provided the expression profile data and bioprediction information of COMMD6 in a variety of tissues, which help to elucidate its functions and clinical values in various human tumours.

## Methods

### Human specimen collection

A total of 100 cases of stomach, kidney, breast, colon, oesophagus, endometrium, placenta, cervical epithelial, liver and lung tissues without presence of tumour cells were collected. In addition, human paired tumour and normal tissues were collected from 25 cases of liver, 20 cases of colon, 15 cases of breast, 10 cases of kidney, 8 cases of stomach, 5 cases of bladder, 5 cases of lung and 5 cases of oesophagus cancer patients who underwent surgical resection in Nanfang Hospital (Guangzhou, China) from January 2016 to January 2017. None of these patients received radiotherapy, chemotherapy, biological immunotherapy or multiple operations before surgery. The collection of tissues for research purpose was approved by the Ethics Committee of the Nanfang Hospital.

### Mice and tissue preparation

Six male BALB/c mice (4 weeks old, 18–20 grams per mouse) were purchased from Central Laboratory of Animal Science at Southern University (Guangzhou, China). Use of laboratory animals were approved by the Institutional Animal Care and Use Committee in Southern Medical University. BALB/c mice were fed with sterile water and fodder in specific pathogen-free (SPF)-class housing of laboratory. All mice were anesthetised in a chamber containing isoflurane and then sacrificed by cervical dislocation. Different organs and tissues were taken for subsequent real-time PCR and immunohistochemistry examination for the detection of COMMD6. The remaining tissues were stored at −80 °C for later use.

### Real-time PCR

Total RNA was extracted from the tissue samples using Trizol reagent (Invitrogen, USA) according to established protocols. The PrimeScript RT Reagent Kit (Perfect Real Time, Takara) was used to generate cDNA for the detection of COMMD6. The expression level of COMMD6 was analysed by quantitative Real-Time PCR (qRT-PCR) using the qRT-PCR Detection Kit (GeneCopeia). The primer sequences of COMMD6 (*Homo sapiens*): Forward: 5′-GGAAACTGGGTATGGCTGTGA-3′, Reverse: 5′-TGTGGAATCGTCATTTC AAAGCA-3′. The primer sequences of COMMD6 (*Mus musculus*): Forward: 5′- CAAGTATCCTTACGTGGCAGTG-3′, Reverse: 5′-TGTGGAATTGTCATCTCG ATGGA-3′. The relative COMMD6 level was calculated using the comparative Ct method (^ΔΔ^Ct).

### Immunohistochemistry (IHC) assay

Tissues were fixed in 4% paraformaldehyde, dehydrated through graded alcohol, embedded in paraffin and cut into 4 μm sections. Sections were deparaffinised using xylene and hydrated through graded alcohol to water. Antigen retrieval was performed by boiling at 100 °C for 10 min in 10 mmol/L citrate buffers (pH = 6.0). Then sections were incubated with COMMD6 antibody (AB42937, ABSci, China) overnight at 4 °C. Then the horseradish-peroxidase-conjugated anti-goat secondary antibody (DakoCytomation, Glostrup, Denmark) was applied and incubated for 1 h at room temperature. The visualisation signal was developed with 3, 3-diaminobenzidine tetra hydrochloride staining, counterstained in haematoxylin, sealed with neutral balsam and observed under microscope (Olympus, Japan). The expression of COMMD6 was calculated as the sum of the percent positivity of stained tumour cells and the staining intensity. The percent positivity was scored as “0,” 0%; “1,” 1–25%; “2,” 26–50%; “3,” 51–75% and “4,” >75%. The staining intensity was scored as “0” (no staining), “1” (weakly stained), “2” (moderately stained) and “3” (strongly stained). Both percent positivity of cells and staining intensity were decided in a double blinded manner. The staining of COMMD6 was assessed as follow: (−) means a final staining score of <3; (+) a final staining score of 3; (++) a final staining score of 4 and (+++) a final staining score of ≥5.

### Phylogenetic analysis

The evolutionary history was inferred using the Neighbor-Joining method. The optimal tree with the sum of branch length = 8.68230306 is shown. The percentage of replicate trees in which the associated taxa clustered together in the bootstrap test (1000 replicates) are shown next to the branches. The tree is drawn to scale, with branch lengths in the same units as those of the evolutionary distances used to infer the phylogenetic tree. The evolutionary distances were computed using the Poisson correction method and are in the units of the number of amino acid substitutions per site. This analysis involved 17 *homo sapiens* amino acid sequences obtained from Uniprot database. All ambiguous positions were removed for each sequence pair (pairwise deletion option). There were a total of 257 positions in the final dataset. Evolutionary analyses were conducted in MEGA X.^23^

### Bioinformatics mining of COMMD6

The chromosome location site of COMMD6 gene was analysed by GeneCards database (https://www.genecards.org/). The gene structure and protein sequence of COMMD6 was analysed in Uniprot database (https://www.uniprot.org/) and drawn with Illustrator for biological sequences software (IBS, http://ibs.biocuckoo.org/). DNAMAN software (LynnonBiosoft, USA) was applied to compare the protein sequence of COMMD6 between human and other species. The expression profile of COMMD6 in various human normal tissues was examined using UCSC database (https://genome.ucsc.edu/). The expression, copy number variation (CNV) and methylation of COMMD6 in human cancer cell lines were analysed using the CCLE database (https://portals.broadinstitute.org/ccle). The mutation of COMMD6 in human tumour cell lines evaluated by COSMIC database (https://cancer.sanger.ac.uk/cosmic). The RNA-sequencing data and patients’ survival of COMMD6 in human tissues based on The Cancer Genome Atlas (TCGA) were analysed using the GEPIA database (http://gepia.cancer-pku.cn/index.html).

MicroRNAs were predicted using miRanda, miRDB, miRwalk, DIANAmT and Targetscan databases. LncRNAs were predicted using DIANA databases. Transcription factors (TFs) of COMMD6 were predicted using the GCBI database (https://www.gcbi.com.cn). The ceRNA network was constructed using Cytoskype software. The heatmaps of differentially expressed microRNAs, LncRNAs, TFs and COMMD6 co-expressing genes were performed using the “limma”, “edgeR”, “pheatmap” and “ggplot2” packages in R version 3.5.3 (The R Foundation for Statistical Computing, Vienna, Austria; http://www.r-project.org/). In addition, the Gene Ontology (GO) enrichment analysis for biological process (BP), cellular component (CC), molecular function (MF) and Kyoto Encyclopedia of Genes and Genomes (KEGG) pathway were generated by R software using “clusterProfiler”, “org.Hs.eg.db”, “enrichplot” and “ggplot2” packages.

### Statistical analysis

All the data were analysed using the SPSS21.0 software (IBM, USA). Student’s *t*-test was applied for the comparison between two groups, and one-way analysis of variance (ANOVA) followed by LSD comparison test was used for the comparison between at least three groups. Nonparametric test was used to analyse the IHC scores of COMMD6 in various tumours. Correlation analysis of COMMD6 was assessed by Pearson correlation method. The *P*-value <0.05 was considered statistically significant.

## Result

### Structure and phylogenetic conservative analysis of COMMD6

**Fig. 1:**
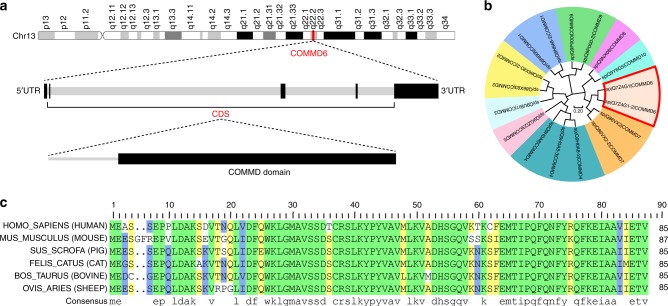
Gene structure and phylogenetic conservative analysis of COMMD6. **a** Chromosome location of COMMD6 (Highlighted in red line) was shown in top panel. Gene structure of COMMD6 was shown in median panel. Black box represents exons and grey box represents introns. Protein structure of COMMD6 was shown in bottom panel (Black box represents COMMD domain). **b** Phylogenetic tree of *Homo sapiens* COMMD family proteins including COMMD6 (Highlighted in red frame). The tree is drawn to scale, with branch lengths in the same units as those of the evolutionary distances used to infer the phylogenetic tree. The evolutionary distances were computed using the Poisson correction method and are in the units of the number of amino acid substitutions per site. **c** Alignments analysis of COMMD6 protein sequence among *Homo sapiens* (UniProtKB-Q7Z4G1), *Mus musculus* (UniProtKB-Q3V4B5), *Sus scrofa* (UniProtKB-A0A286ZXE0), *Felis catus* (UniProtKB-M3WUJ0), *Bos taurus* (UniProtKB-Q2KIY) and *Ovis aries* (UniProtKB-W5Q264). The degree of conservation of each amino acid residue among these sequences is indicated green (100% conserved), yellow (75% conserved) and blue (50% conserved)

COMMD6, a member of the COMMD family, was located at 13q22.2. Gene structure of COMMD6 included 5′UTR exon, three CDS exons, 3′UTR exon and four introns. The protein structure of COMMD6 is characterised by the presence of copper metabolism gene MURR1 domain (COMMD) (Fig. 1a). To explore the conservative of COMMD6 during molecular and species evolution, we compared the protein sequences encoded by COMMD6 with other COMMD family proteins and mammal species. Phylogenetic analysis results showed that all *homo sapiens* COMMD family members were divided into three clusters. Cluster1 included COMMD2, COMMD3, COMMD4 and COMMD5. COMMD6, as well as COMMD7, COMMD8 and COMMD10, was classified into cluster2. Cluster3 included COMMD1 and COMMD9 (Fig. 1b). Sequence comparison showed that *Homo sapiens* COMMD6 shared 84, 85, 88, 88 and 87% identity to *Mus musculus, Sus scrofa*, *Felis catus*, *Bos taurus* and *Ovis aries,* respectively, suggesting that COMMD6 is highly conserved in mammals (Fig. 1c).

### Biological distribution and expression of COMMD6 in BALB/c mice tissues

**Fig. 2:**
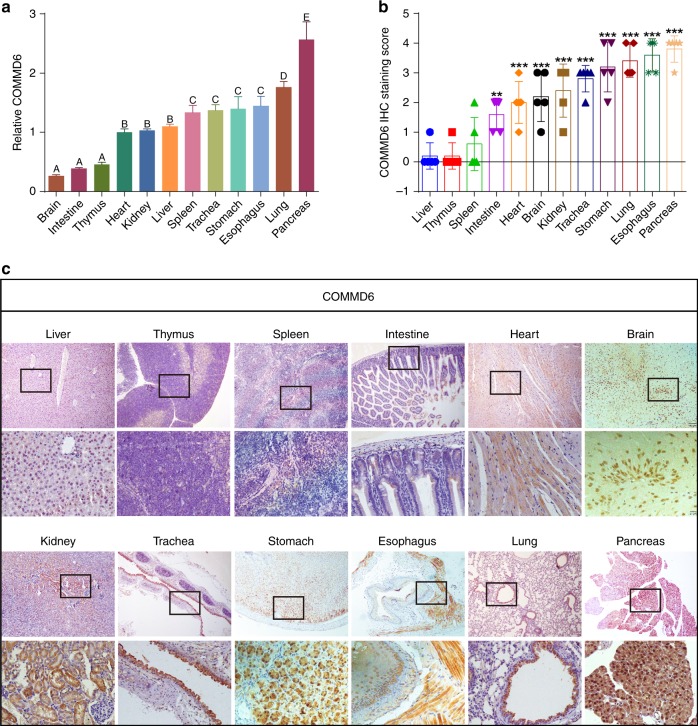
Biological distribution and expression of COMMD6 in normal tissues of BALB/c mice and human. **a** Expression of COMMD6 mRNA in various tissues of BALB/c mice by qPCR assay. The different letters stand for significant difference (*P* < 0.01), while tissues with the same letter show no difference (*P* > 0.05). All samples were tested in triplicate. **b**, **c** Expression and distribution of COMMD6 proteins in human normal tissues by IHC. Each bar represents the mean ± SD; ***P < 0.001

BALB/c mice are the most commonly used laboratory animals to investigate the mechanism of tumour development and progression. We firstly detected the mRNA expression of COMMD6 in 12 different types of tissue samples derived from six BALB/c mice. As shown in Fig. 2a, COMMD6 mRNA was expressed in all detected tissues examined by qPCR analysis. The different letters stand for significant difference (*P* < 0.01), while tissues with the same letter show no difference (*P* > 0.05). The relative expression of COMMD6 mRNA in pancreas was significantly higher than that in other tissues. Lung tissues displayed higher COMMD6 mRNA level than that in oesophagus, stomach, trachea and spleen tissues (*P* < 0.05). The expression of COMMD6 mRNA in spleen is higher than that in liver, kidney and heart. The expression of COMMD6 mRNA in brain, intestine and thymus tissues was significantly lower than that compared to other tissues (*P* < 0.05).

The expression levels and distribution of COMMD6 were further examined in BALB/c mice tissues by immunohistochemistry (IHC) assay. As shown in Fig. 2b, c, varying expression levels of COMMD6 were found in different types of tissues. In the pancreas, strong positive staining of cytoplasm and nucleus was found in acinar epithelial cells. In the lung, bronchial epithelium exhibited strong positive staining in cytoplasm and nucleus, while no staining was found in alveolar epithelial cells. In oesophagus, squamous epithelial cells showed moderate to strong positive staining in nucleus while strong positive staining was found in cytoplasm of smooth muscle cells. Moderate to strong staining was found in cytoplasm and nucleus of gastric gland cells. Renal tubular epithelial cells showed strong staining in cytoplasm while no staining was detected in glomerular epithelial cells. Tracheal mucosa exhibited strong staining in the cytoplasm, while the chondrocytes of hyaline cartilage showed moderate positive staining in the cytoplasm. Brain neurons showed strong staining while cardiac muscle fibres exhibited weak staining in the cytoplasm. Glandular epithelial cells of the intestine exhibited weak staining in the cytoplasm. No obvious staining of COMMD6 was detected in the spleen, thymus and liver tissues by IHC.

### Biological distribution and expression of COMMD6 in human normal tissues

**Fig. 3:**
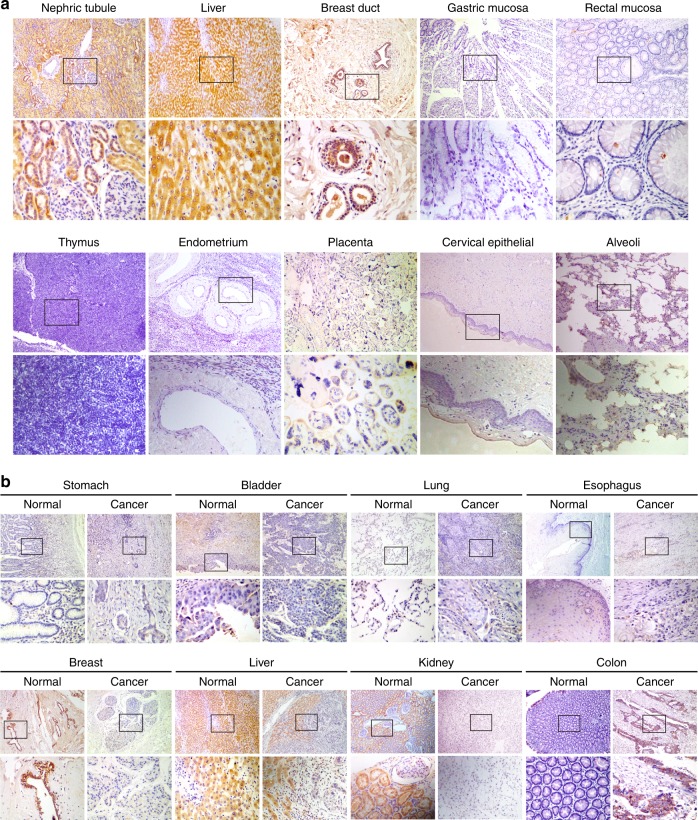
Biological distribution and expression of COMMD6 in human normal and tumour tissues. **a** Expression and distribution of COMMD6 proteins in various human normal tissues analysed by IHC assay. **b** Expression and distribution of COMMD6 proteins in paired human tumours and normal tissues examined by IHC assay

To explore the biological distribution and expression of COMMD6 in human normal tissues, we detected COMMD6 in 10 different types of human normal tissues using IHC assay (Fig. 3a). COMMD6 was strongly expressed in the cytoplasm of renal tubular epithelial cells. In the liver, COMMD6 staining was strongly positive in the cytoplasm of hepatocytes, and some of it was expressed in the nucleus. In the breast, COMMD6 was strongly expressed in the cytoplasm of luminal epithelial cells. No staining of COMMD6 protein was found in human gastric mucosa, colon mucosa, thymus, endometrium, placenta, cervical epithelium and alveoli tissues.

Furthermore, the expression profile of COMMD6 in various human normal tissues was further examined using UCSC database (Figure S1). The expression and distribution of COMMD6 was similar to the above mentioned IHC results. COMMD6 mRNA was highly expressed in human breast tissues and kidney cortex tissues. Paradoxically, COMMD6 mRNA was lowly expressed in liver tissues, which was highly expressed in liver specimens detected by IHC assay. The level of COMMD6 mRNA was moderately expressed in colon, lung and stomach tissues, which were negatively expressed in corresponding tissues detected by IHC assay.

### Expression, copy number variation (CNV), methylation and mutation of COMMD6 in human tumours

To explore the role of COMMD6 in human tumour cell lines, COMMD6 expression was firstly evaluated using Cancer Cell Line Encyclopedia (CCLE) database. Both mRNA expression and CNV of COMMD6 were highest in acute myelocytic leukaemia (AML), meningioma, colorectal cancer and diffuse large B-cell lymphoma (DLBCL) cell lines (Fig. S2A, B). In addition, the methylation status of COMMD6 was highest in lymphocyte malignancies, and lowest in soft tissue cancer, osteosarcoma, leukaemia, giant cell tumour, Ewing’s sarcoma and oesophagus cancer cell lines (Fig. S2C). COSMIC database analysis showed that COMMD6 was highly mutated in skin cancer, soft tissue cancer and melanoma cells. In contrast, rare mutation of COMMD6 was observed in cervical, colon and rectal cancer cells (Fig. S3). The expression level of COMMD6 mRNA was further exhibited in 31 types of human paired tumour and normal tissues using GEPIA database. The median expression of COMMD6 mRNA was higher in 20 types of human tumours compared to corresponding normal tissues, such as colorectal cancer, brain lower grade glioma and acute myeloid leukaemia. Eleven types of tumours expressed lower COMMD6 compared to normal tissues, especially in adrenocortical carcinoma, pheochromocytoma and paraganglioma and ovarian cancer (Fig. S4A, B).

We further detected the expression of COMMD6 protein in eight types of human paired tumour and normal tissues by IHC assay (Fig. 3b). COMMD6 protein was negatively expressed in both tumour tissues and corresponding normal tissues of stomach, bladder, lung and oesophagus. In contrast, COMMD6 was highly expressed in the cytoplasm of human breast, liver and kidney normal tissues while negatively expressed in corresponding tumour tissues. In colon, COMMD6 was highly expressed in the cytoplasm of tumour tissues while negatively expressed in normal mucosa. Moreover, the expression pattern of COMMD6 in liver and kidney by IHC assay was consistent with the GEPIA results. COMMD6 expression in BRCA is a bit higher than that of normal tissues by GEPIA, which was contrary to that observed by IHC staining.

### The clinical prognosis significance of COMMD6 in human tumours

To further evaluate the prognostic value of COMMD6, we analysed the correlation between COMMD6 expression and patient’s survival using GEPIA database. High expression of COMMD6 was associated with shorter overall survival (OS) and disease free survival (DFS) in patients with head and neck squamous cell carcinoma (HNSC, *P* = 0.0084 and HR = 1.7 for OS; *P* = 0.0056 and HR = 2.0 for DFS), cholangiocarcinoma (CHOL, *P* = 0.05 and HR = 2.7 for OS; *P* = 0.029 and HR = 2.8 for DFS) and adrenocortical carcinoma (ACC, *P* = 0.0077 and HR = 2.9 for OS; *P* = 0.0053 and HR = 2.6 for DFS) (Fig. S5A–F). In contrast, high expression of COMMD6 was associated with longer OS and DFS in patients with brain lower grade glioma (LGG, *P* = 0.0016 and HR = 0.56 for OS; *P* = 0.0096 and HR = 0.66 for DFS) and uveal melanoma (UVM, *P* = 0.0085 and HR = 0.30 for OS; *P* = 0.028 and HR = 0.35 for DFS) (Fig. S6A–D). In addition, high expression of COMMD6 was associated with longer OS in patients with testicular germ cell tumours (TGCT, *P* = 0.05, HR = 1.7E-09), longer DFS in patients with thyroid carcinoma (THCA, *P* = 0.017, HR = 0.48) and uterine corpus endometrial carcinoma (UCEC, *P* = 0.029, HR = 0.47) (Fig. S6E–G). These data highlight the prognostic values of COMMD6 in human tumours.

### Construction of a ceRNA network involved in human HNSC

**Fig. 4:**
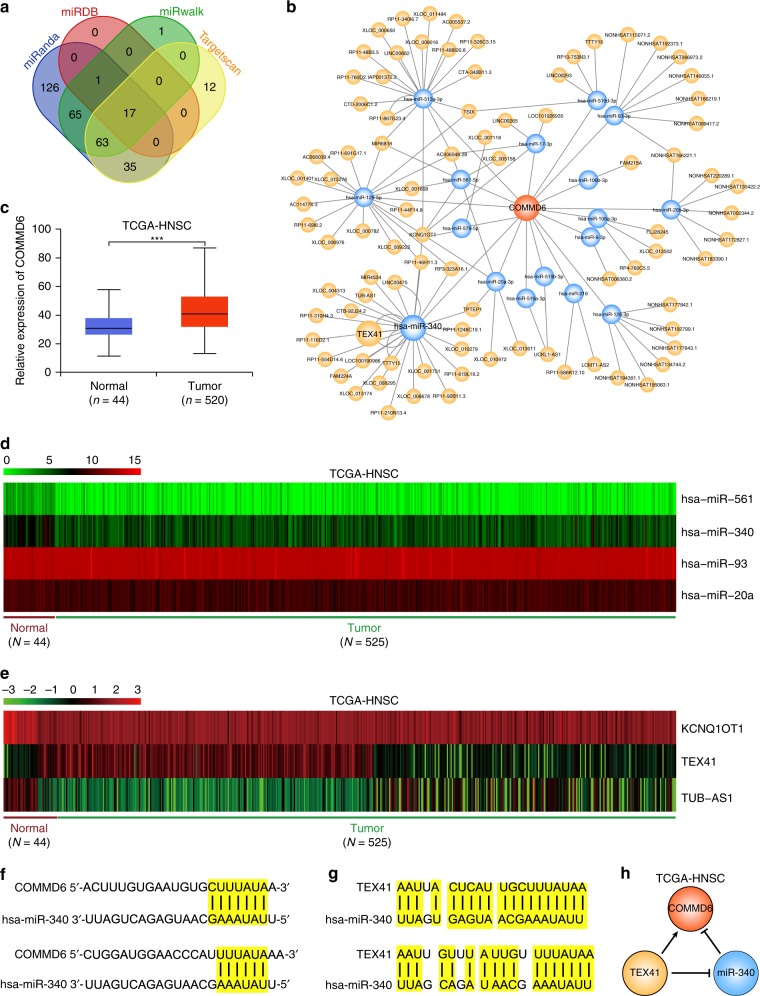
Construction of a ceRNA network involved in HNSC. **a** Prediction of upstream miRNAs of COMMD6 using miRanda, miRDB, miRwalk and Targetscan databases. **b** Construction of lncRNA-miRNA-COMMD6 network. **c** Expression of COMMD6 in HNSC derived from TCGA database. **d**, **e** Differentially expressed miRNAs (**d**) and lncRNAs (**e**) in the network between normal tissues and tumour in HNSC derived from TCGA database. **f**, **g** Complementary sequences between hsa-miR-340 and COMMD6 and TEX41. **h** A ceRNA network formed by TEX41, miR-340 and COMMD6 in HNSC

To explore the upstream molecular mechanisms that modulate COMMD6 expression in human cancer, we constructed a potential competing endogenous RNA (ceRNA) network, which has been reported to be a new and complex regulatory network involved in many diseases including cancer.^24^ Firstly, 17 upstream microRNAs (miRNAs) of COMMD6 were predicted using miRanda, miRDB, miRwalk and Targetscan databases, such as has-miR-561 and has-miR-340 (Fig. 4a). Then we predicted lncRNAs based on the interaction of these miRNAs and constructed lncRNA-miRNA-COMMD6 ceRNA network (Fig. 4b).

To validate the prediction results, the expression levels of COMMD6 and the differentially predicted miRNAs and lncRNAs in the network were compared between normal and tumour samples in human HNSC obtained from TCGA database. A significantly higher level of COMMD6 was found in tumours compared to normal tissues (Fig. 4c), indicating a tumour promoting role of COMMD6 in HNSC. Moreover, tumour expressed higher hsa-miR-93 and hsa-miR-20a while lower hsa-miR-561 and hsa-miR-340 compared to normal tissues (Fig. 4d, *P* < 0.01). LncRNAs KCNQ1OT1 and TUB-AS1 were lower while TEX41 was higher in tumours than those in normal tissues (Fig. 4e, *P* < 0.001).

The above results indicate that these differentially expressed genes might form network involved in HNSC. Indeed, we found the complementary sequences between the 3′ untranslational region of COMMD6 and hsa-miR-340 (Fig. 4f). Moreover, complementary sequences were detected between lncRNA TEX41 and hsa-miR-340 (Fig. 4g). Based on the differential expression and complementary results, lncRNA TEX41 might promote COMMD6 expression by inhibiting the expression of has-miR-340 in HNSC (Fig. 4h).

### Construction of transcriptional network of COMMD6 in CHOL

**Fig. 5:**
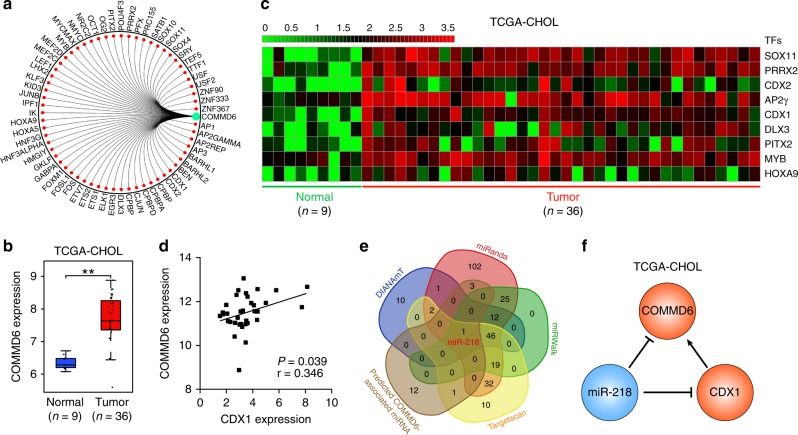
Construction of transcriptional network of COMMD6 in CHOL. **a** Prediction of transcription factors (TFs) in COMMD6 promoter using GCBI online database. **b** Expression of COMMD6 in CHOL derived from TCGA database. **c** Differentially expressed TFs between normal and tumour tissues in CHOL derived from TCGA database. **d** Pearson analysis of the correlation between the expression of COMMD6 and CDX1 in CHOL derived from TCGA database. **e** Venn map of the predicted overlapping miRNAs involved in the regulation of COMMD6 and CDX1. **f** A transcriptional network formed by miR-218, CDX1 and COMMD6 in CHOL derived from TCGA database

To further explore the potential upstream mechanism of COMMD6 involved in tumour progression, we presented a transcriptional regulatory network and 62 transcription factors (TFs) that participate in the regulation of COMMD6 expression (Fig. 5a). This regulatory network was further verified in CHOL. As shown in Fig. 5b, COMMD6 was significantly higher in tumour tissues compared to normal tissues. Consistently, nine TFs were significantly higher in tumour compare to normal tissues (Fig. 5c, *P* < 0.01). Furthermore, Pearson analysis showed a positive correlation between the expression of CDX1 and COMMD6 in CHOL (Fig. 5d, *P* = 0.039, r = 0.346). None of the other eight TFs significantly correlated with COMMD6 in CHOL (Fig. S7, *P* > 0.05). Moreover, we predicted the upstream miRNAs of CDX1 using DIANAmT, miRanda, miRWalk and Targetscan databases and compared them with the 17 miRNAs upstream of COMMD6. MiR-218 was the only overlapping miRNA in our prediction system (Fig. 5e). Given these above results, we proposed that COMMD6 could be directly inhibited by miR-218 or indirectly inhibited by miR-218 mediated suppression of CDX1 in CHOL (Fig. 5f).

### Biological enrichment analysis of COMMD6 downstream pathway

**Fig. 6:**
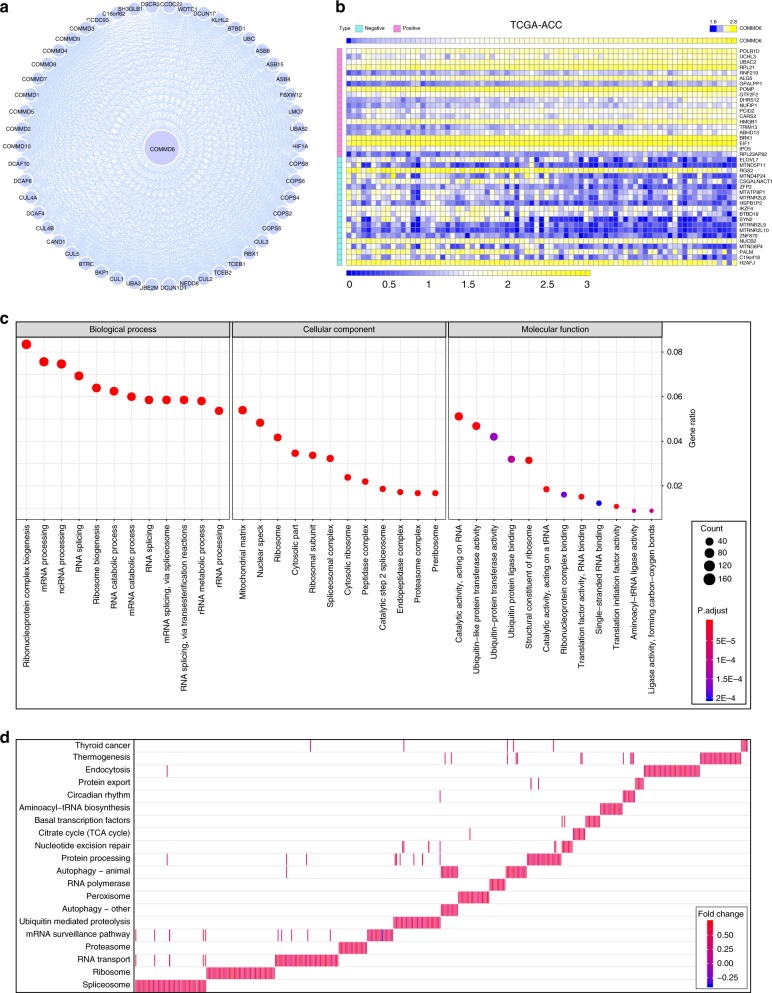
Biological enrichment analysis of COMMD6 downstream pathway. **a** The interacting proteins of COMMD6 predicted with string database. **b** Co-expressing genes of COMMD6 in ACC derived from TCGA exhibited by heatmap. **c** Annotation of the biological function of co-expressing genes of COMMD6 at three levels of biological process, cellular component and molecular function. **d** KEGG pathway enrichment analysis of the co-expressing genes of COMMD6

To explore the downstream pathway of COMMD6, we investigated the interacting proteins of COMMD6 using string database (Fig. 6a). Moreover, the potential roles of COMMD6 and its interacting genes were further explored using DAVID database. Gene ontology analysis showed that they were mainly members of the ubiquitin ligase complex and associated with post-translational protein modification, which participated in the regulation of ubiquitin protein ligase binding (Fig. S8A–C). Furthermore, the potential signalling pathways associated with COMMD6 and its interacting genes were predicted using KEGG pathway database. Results showed that they closely related to the regulation of ubiquitin mediated proteolysis signalling pathway (Fig. S8D).

We further explored the potential roles of COMMD6 co-expressing genes in human ACC derived from TCGA database. The top 20 positive co-expressing genes, as well as the top twenty negative co-expressing ones of COMMD6 were shown in Fig. 6b. GO analysis showed that these genes were mainly located in mitochondrial matrix and nuclear speck, which may execute catalytic activity and ubiquitin-like protein transferase activity and participate ribonucleoprotein complex biogenesis and mRNA processing biological process (Fig. 6c). KEGG analysis showed that these genes were most likely associated with spliceosome and ribosome (Fig. 6d, S9A, B).

## Discussion

Cancer is the malignant disease that severely threatens human health and the incidence and mortality are rapidly growing worldwide.^1^ Aberrant activation of the NF-κB signalling pathway has been observed in many human cancers,^22^ making it a promising therapy target for tumour. COMMD6 has been reported to completely abolish NF-κB pathway activity by the mutation of the amino acid residues Trp24 and Pro41 in the COMM domain in HEK293 cells, indicating that COMMD6 may also play a vital role in the tumorigenesis and malignant progression. However, rare evidence has been reported about the role of COMMD6 in tumours. Here, we provided the tissue-specific expression, biology function and the possible mechanism of COMMD6 in a variety of tissues, aiming to elucidate its functions and clinical values in human tumours.

In this study, COMMD6 was highly conserved in mammals, indicating its potential role in the evolution of species. Phylogenetic analysis results showed that COMMD6 was clustered with COMMD7, COMMD8 and COMMD10 rather than other COMMD family members, indicating that these four COMMD members might play familiar roles during evolution. Moreover, it was established that COMMD7 promoted migration and invasion of hepatocellular carcinoma^13^ and our previous study found that COMMD10 inhibited metastasis of colorectal cancer,^17^ reflecting the potential similar role of COMMD6 in human cancer.

As the most commonly used laboratory animals to investigate human tumours, BALB/c mice were further investigated for the expression and distribution of COMMD6. Both mRNA and protein levels of COMMD6 were consistently highly expressed in pancreas, lung, oesophagus, stomach and trachea tissues while lowly expressed in thymus. The wide expression of COMMD6 indicates its important role in the development of BALB/c mice tissues. In contrast, brain tissues expressed the lowest mRNA but relatively higher protein levels of COMMD6. We speculated that the difference in the expression level of COMMD6 between mRNA and protein might due to epigenetic^21^ or post-translational modification^25^ of COMMD6, which remains to be investigated.

We further analysed the expression of COMMD6 in human tumours by using GEPIA database. Ten tumours expressed higher level, while 21 tumours showed lower level of COMMD6 mRNA compared to corresponding normal counterparts, implying that different mechanisms are involved in the regulation of COMMD6 expression in the development of human tissues. Furthermore, the expression of COMMD6 in eight kinds of tumours and matched normal specimens were examined using IHC to verify the GEPIA result. However, there were some differences in the expression of COMMD6 in tumour and normal tissues between GEPIA database and our IHC results. We speculated that the data of GEPIA derived from TCGA database was characterised by global ethnicity. The ethnic difference may cause completely different biological characteristics, leading to tumour heterogeneity. In addition, the small size of our specimens may also lead to bias in the expression of COMMD6. More importantly, the expression of COMMD6 was associated with the survival of cancer patients. High expression of COMMD6 correlates with shorter survival in HNSC, CHOL, ACC patients and longer survival in LGG, UVM, TGCT, THCA and UCEC patients, indicating that the expression of COMMD6 may be helpful in understanding the pathological mechanism and providing new diagnostic and therapeutic biomarkers for these tumours.

CeRNAs (lncRNAs and circRNAs) have been reported to indirectly modulate mRNAs via regulating overlapping microRNAs, which represents a novel layer of RNA crosstalk and participates in the development and progression of human tumours.^24^ In our study, we firstly constructed a ceRNA network based on miRNAs-COMMD6 and miRNAs-LncRNAs interactions to analyse the upstream mechanism involved in the regulation of COMMD6 expression. In addition, the TEX41-miR-340-COMMD6 network was further verified based on the differential expression profiles in HNSC. It has been well established that miR-340 acts as a tumour suppressor in a wide range of human tumours. In non-small cell lung cancer, low miR-340 expression predicts poor prognosis and promotes cell proliferation by targeting CDK4.^26^ In colorectal cancer, miR-340 inhibits growth and increases chemosensitivity of cancer cells by targeting RLIP76.^27^ Similarly, miR-340 inhibits the metastasis of cervical cancer by targeting EphA3.^28^ MiR-340 was also reported to inhibit the development of osteosarcoma by downregulating the Wnt/β-catenin pathway.^29^ In glioma, miR-340 was defined as a tumour suppressor and inhibited glioma-initiating cells mediated tumorigenesis by targeting tissue plasminogen activator.^30^ What’s more, lncRNA TEX41 was a hotspot gene integrated by HPV in the RNA samples of cervical cancer, suggesting a tumour promoting role of TEX41 in cervical cancer.^31^ In our study, the expression of miR-340 in HNSC was lower compared to normal tissues. Both TEX41 and COMMD6 were higher in HNSC than those in normal tissues. These results imply a potential tumour suppressing role of miR-340 and tumour promoting function of TEX41 and COMMD6 in HNSC. Combined with the literature review, the expression and complementary sequences between miR-340 and COMMD6 as well as TEX41, we infer that they may form a TEX41-miR-340-COMMD6 ceRNA network to play an important role in HNSC, but further experimental proof is still needed.

In addition, we constructed a miR-218-CDX1-COMMD6 transcription network to analyse the upstream modulation mechanism of COMMD6 in CHOL. It is found that miR-218 inhibits tumour angiogenesis in gastric cancer by targeting ROBO1.^32^ In lung cancer, miR-218 decreases cell proliferation and invasion by targeting IL6/STAT3 and correlates with poor prognosis.^33^ More importantly, miR-218 inhibits epithelial to mesenchymal transition and increases oxaliplatin intracellular accumulation by suppressing Bmi1 in colorectal cancer.^34^ MiR-218 was lowly expressed in hepatocellular carcinoma, and overexpression of miR-218 suppresses growth, EMT and metastasis of hepatocellular cancer by inhibiting HOXA10, RET and SERBP1.^35–37^ CDX1, a member of the caudal-related homeobox transcription factor gene family, was significantly higher in gastric cancer than normal tissues.^38^ In contrast, CDX1 exerts tumour suppressing role in a wide range of tumours, such as colon, breast and hepatocellular cancer.^39–41^ To date, the expression of CDX1 in CHOL remains unknown. Here, we found that CDX1 was significantly higher in tumour compared to that in normal tissues by analysing the CHOL data obtained from TCGA database. Consistently, as a transcription target of CDX1, COMMD6 was also higher in CHOL than that in normal tissues. What’s more, bioinformatics analysis showed that both COMMD6 and CDX1 were downstream targets of miR-218. Combined with the above expression and correlation analysis, we infer that miR-218-CDX1-COMMD6 network might be involved in the regulation of CHOL, which needs further experiments to verify in future.

By bioinformatics analysis, we found that the roles of COMMD6 were mainly enriched in protein ubiquitination activity besides its binding activity with NF-κB. Moreover, the ubiquitination of the NF-κB has been the most extensively studied functions among the COMMD domain containing family of proteins. For example, COMMD1 binds to an ubiquitin ligase containing Elongins B and C, Cul2 and SOCS1 to accelerate the ubiquitination and degradation of NF-κB subunits.^42^ What’s more, COMMD1 phosphorylates RelA at Ser468 to promote the ubiquitination and degradation of RelA, leading to inhibition of nuclear translocation and inactivation of NF-κB pathway.^43,44^ In addition, it has also been found that COMMD8 interacts with CCDC22 to direct the ubiquitination and degradation of IκB proteins.^45^ Moreover, we previously found that COMMD10 inhibited the metastasis of colorectal cancer by promoting ubiquitination and degradation of NF-κB.^17^ Similarly, we speculated that COMMD6 might be involved in tumours by modulating the ubiquitination and degradation of NF-κB subunits.

COMMD6 was further found to be involved in the regulation of ribonucleoprotein complex biogenesis, RNA processing and splicing process in ACC. Ribosome biogenesis and protein synthesis are crucial cellular processes necessary for sustained cancer cell growth. Mutations of ribosome genes have been detected in various tumours, such as endometrial cancer, colorectal cancer and glioma.^46–48^ Spliceosome has been discovered as a target of oncogenic stress in MYC-driven cancer cells. Inhibition of the spliceosome impairs survival, tumorigenicity and metastasis of MYC-dependent breast cancer.^49^ Therefore, we inferred that COMMD6 may regulate tumorigenesis and development through ribonucleoprotein and spliceosome complex biogenesis.

In conclusion, our study firstly illuminated the expression profile of COMMD6 in BALB/c mice and primary human normal and tumour tissues. More importantly, the expression of COMMD6 could predict the survival of cancer patients, indicating the significant role of COMMD6 in tumour development and progression. Furthermore, we shed light on the underlying tumour promoting role and the mechanism of COMMD6 by constructing a TEX41-miR-340-COMMD6 ceRNA network in HNSC and miR-218-CDX1-COMMD6 transcriptional network in CHOL. In addition, we found that COMMD6 may modulate the ubiquitination and degradation of NF-κB subunits and regulate ribonucleoprotein and spliceosome complex biogenesis in tumours. This study may help to elucidate the functions and mechanisms of COMMD6 in various human tumours, providing a potential biomarker for tumour prevention and therapy.

## Supplementary information

Supplementary material

## Data Availability

All the analysis data were obtained from TCGA database (https://portal.gdc.cancer.gov/).
