# Supplementary material for: Prognosis and modulation mechanisms of COMMD6 in human tumours based on expression profiling and comprehensive bioinformatics analysis
Source: Br J Cancer. 2019 Sep 16;121(8):699–709. doi: 10.1038/s41416-019-0571-x (PMC6889128; doi:10.1038/s41416-019-0571-x)
Supplement: Supplementary file 1 — Supplementary material [file 41416_2019_571_MOESM1_ESM.docx]

**Supplementary material contains nine figures and corresponding figure legends.**

**
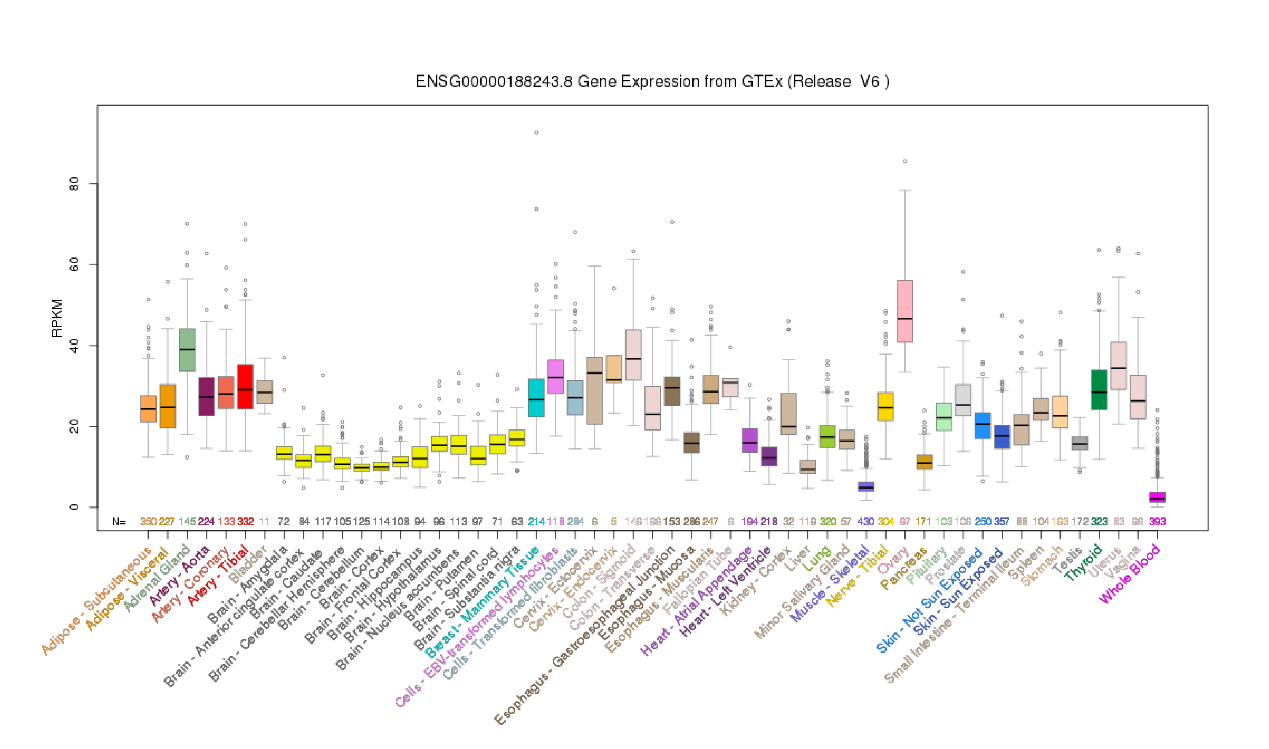
**

**Figure S1 Relative expression of COMMD6 mRNA in various human normal tissues analyzed by UCSC online database.**

**
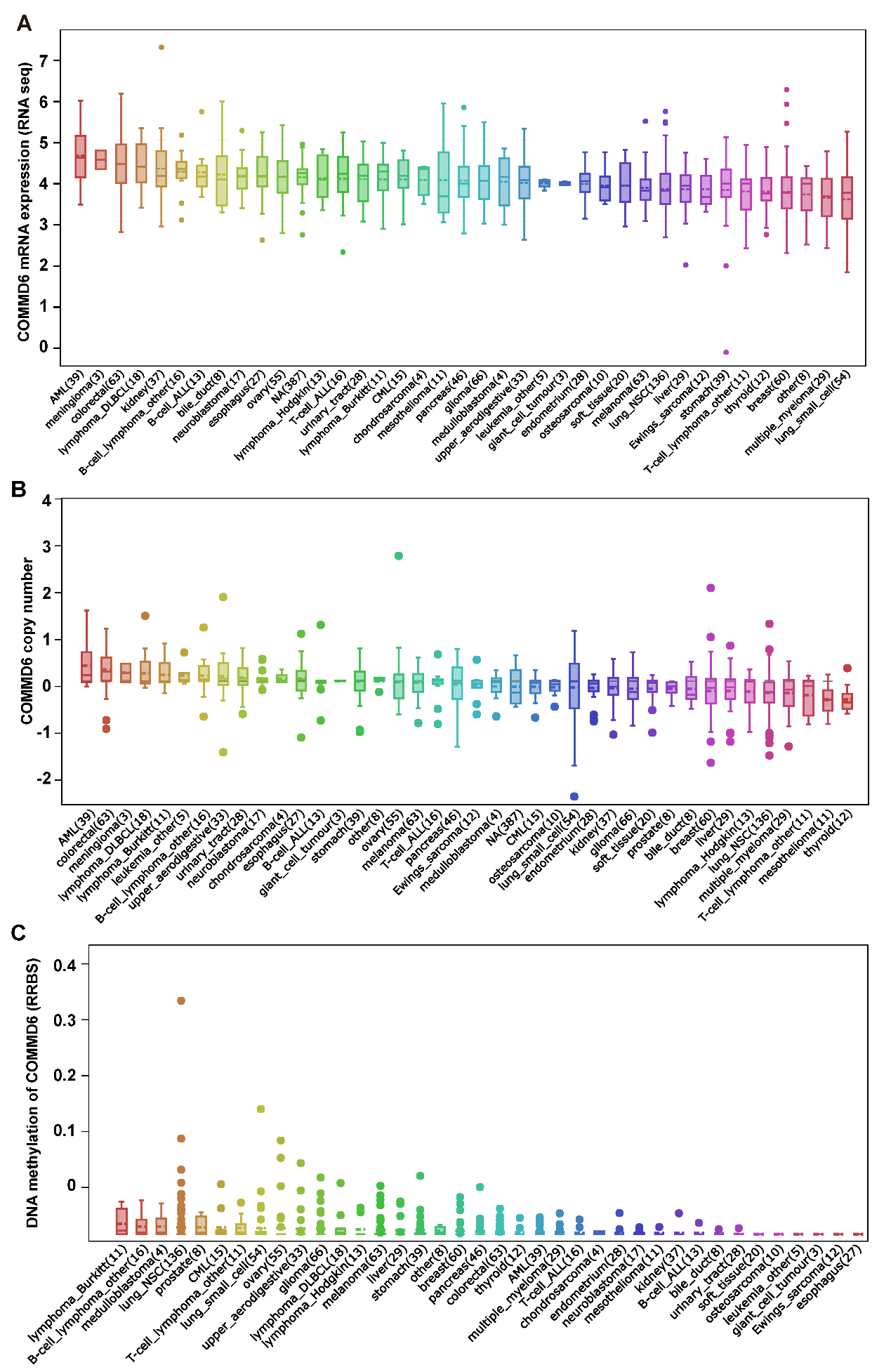
**

**Figure S2 Expression, copy number and DNA methylation of COMMD6 in human cancer cell lines** **analyzed by CCLE database.**


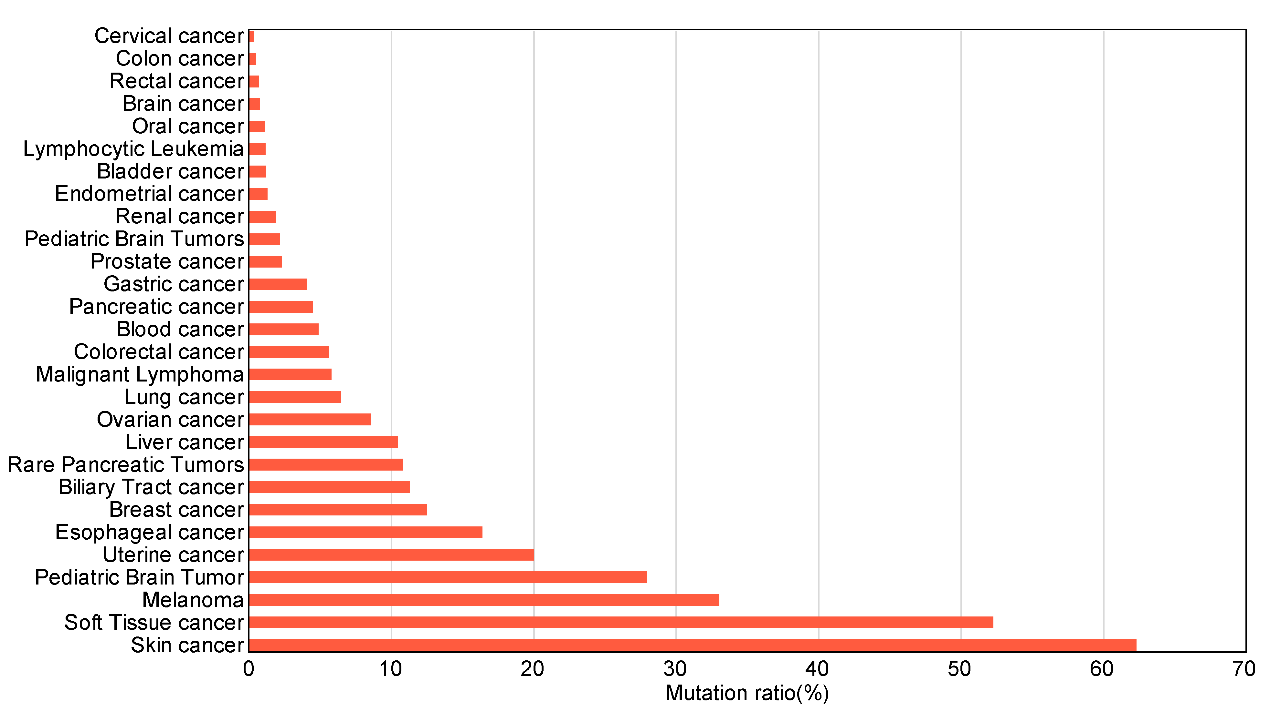


**Figure S3 Mutation of COMMD6 in human various tumors evaluated by COMSIC database.**

**
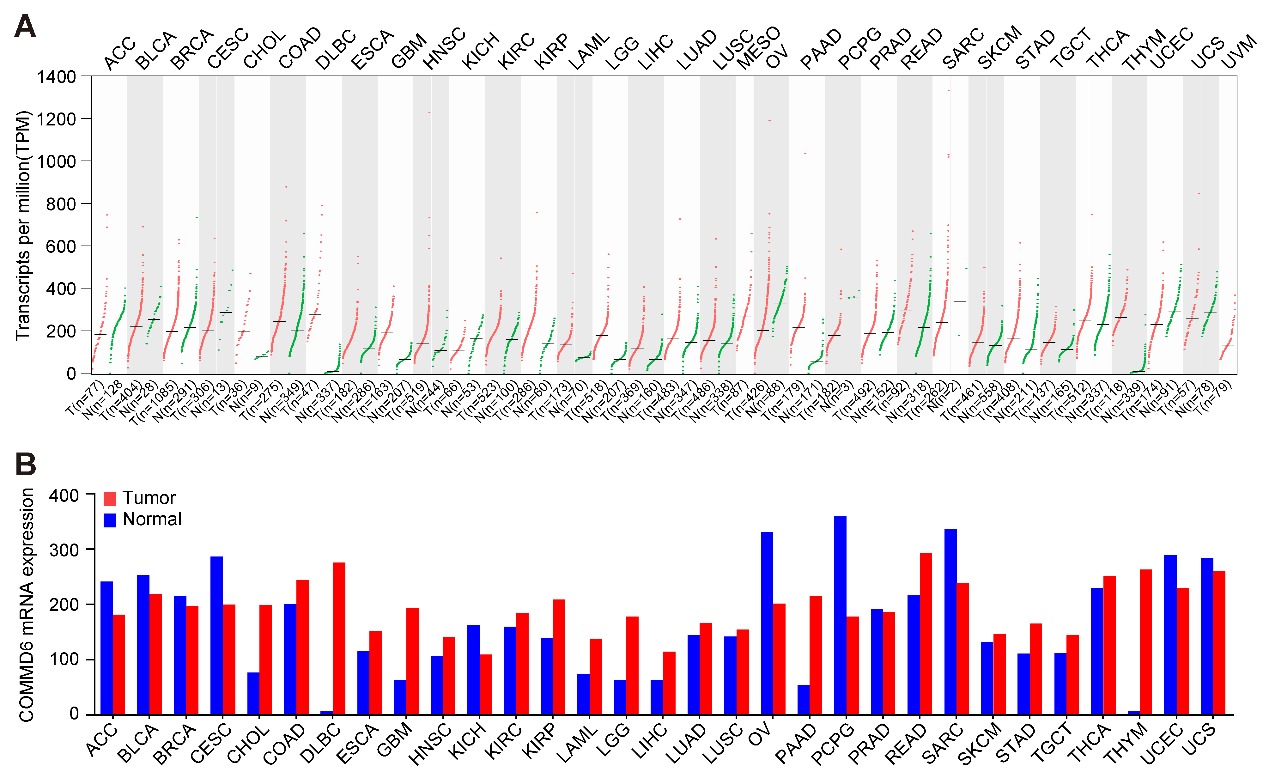
**

**Figure S4 COMMD6 expression in human paired tumor tissues and normal tissues.** **(A)** COMMD6 mRNA expression in human paired normal tissues and tumor tissues analyzed by GEPIA database. **(B)** The median value of COMMD6 mRNA expression in human paired normal tissues and tumor tissues analyzed by GEPIA database. **Abbreviations:** ACC, adrenocortical carcinoma; BLCA, bladder urothelial carcinoma; BRCA, breast invasive carcinoma; CESC, cervical squamous cell carcinoma and endocervical adenocarcinoma; CHOL, cholangiocarcinoma; COAD, colon adenocarcinoma; DLBC, diffuse large B-cell lymphoma; ESCA, esophageal carcinoma; GBM, glioblastoma multiforme; HNSC, head and neck squamous cell carcinoma; KICH, kidney chromophobe; KIRC, kidney renal clear cell carcinoma; KIRP, kidney renal papillary cell carcinoma; LAML, acute myeloid leukemia; LGG, brain lower grade glioma; LIHC, liver hepatocellular carcinoma; LUAD, lung adenocarcinoma; LUSC, lung squamous cell carcinoma; MESO, mesothelioma; OV, ovarian serous cystadenocarcinoma; PAAD, pancreatic adenocarcinoma; PCPG, pheochromocytoma and paraganglioma; PRAD, prostate adenocarcinoma; READ, rectum adenocarcinoma; SARC, sarcoma; SKCM, skin cutaneous melanoma; STAD, stomach adenocarcinoma; TGCT, testicular germ cell tumors; THCA, thyroid carcinoma; THYM, thymoma; UCEC, uterine corpus endometrial carcinoma; UCS, uterine carcinosarcoma; UVM, uveal melanoma.

**
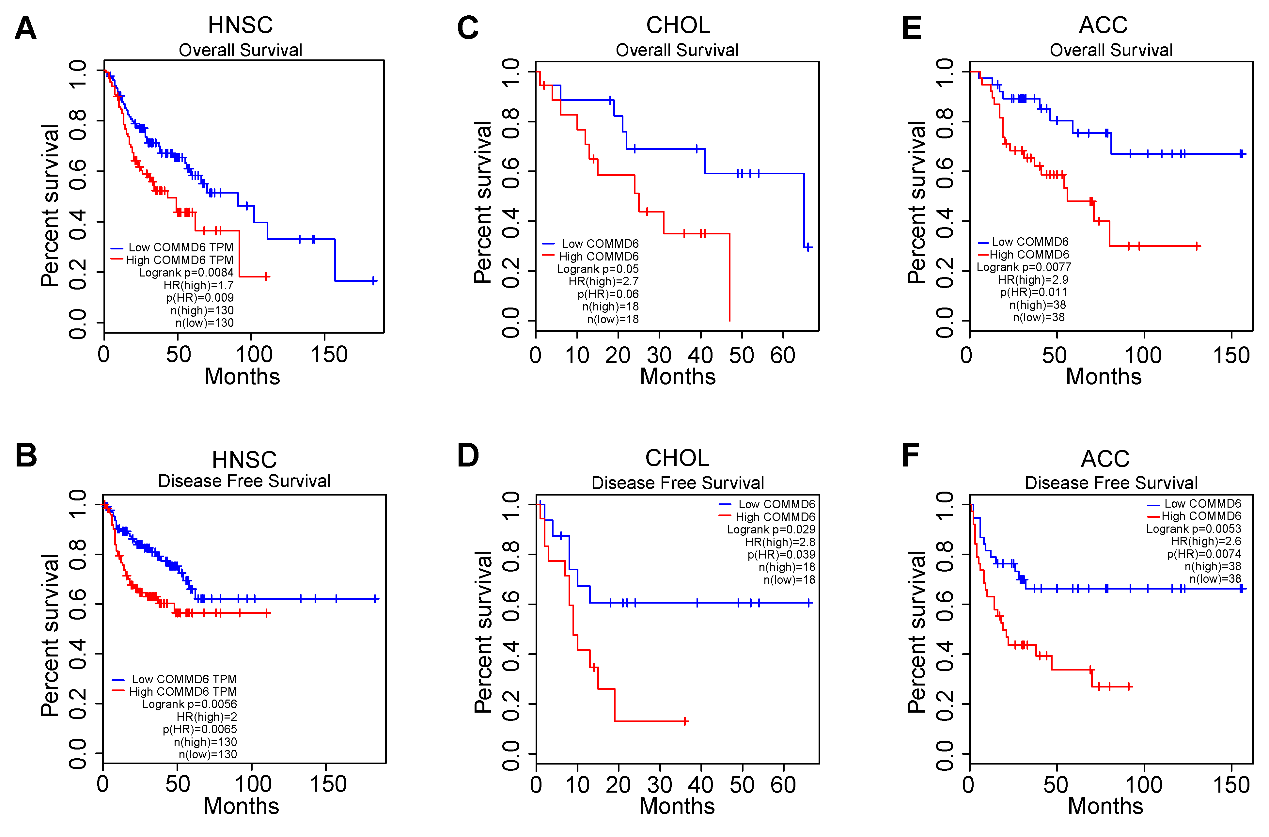
**

**Figure S5 The clinical prognosis significance of COMMD6 in human tumors. (A-F)** Kaplan-Meier analysis of the overall survival (OS) and disease free survival (DFS) in patients with head and neck squamous carcinoma (HNSC), cholangiocarcinoma (CHOL) and adrenocortical carcinoma (ACC) using GEPIA online database.

**
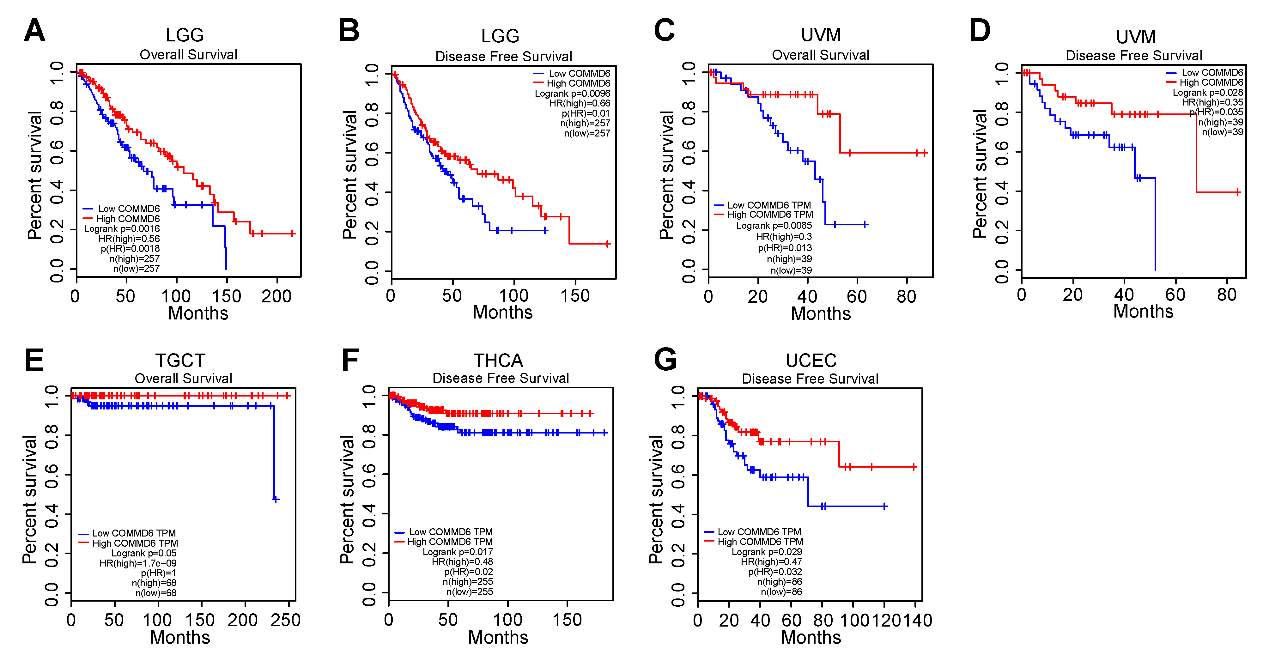
**

**Figure S6 The clinical prognosis significance of COMMD6 in human tumors. (A-G)** Kaplan-Meier analysis of the OS or/and DFS in patients with brain lower grade glioma (LGG), uveal melanoma (UVM), testicular germ cell tumors (TGCT), thyroid carcinoma (THCA) and uterine corpus endometrial carcinoma (UCEC) using GEPIA online database.

**
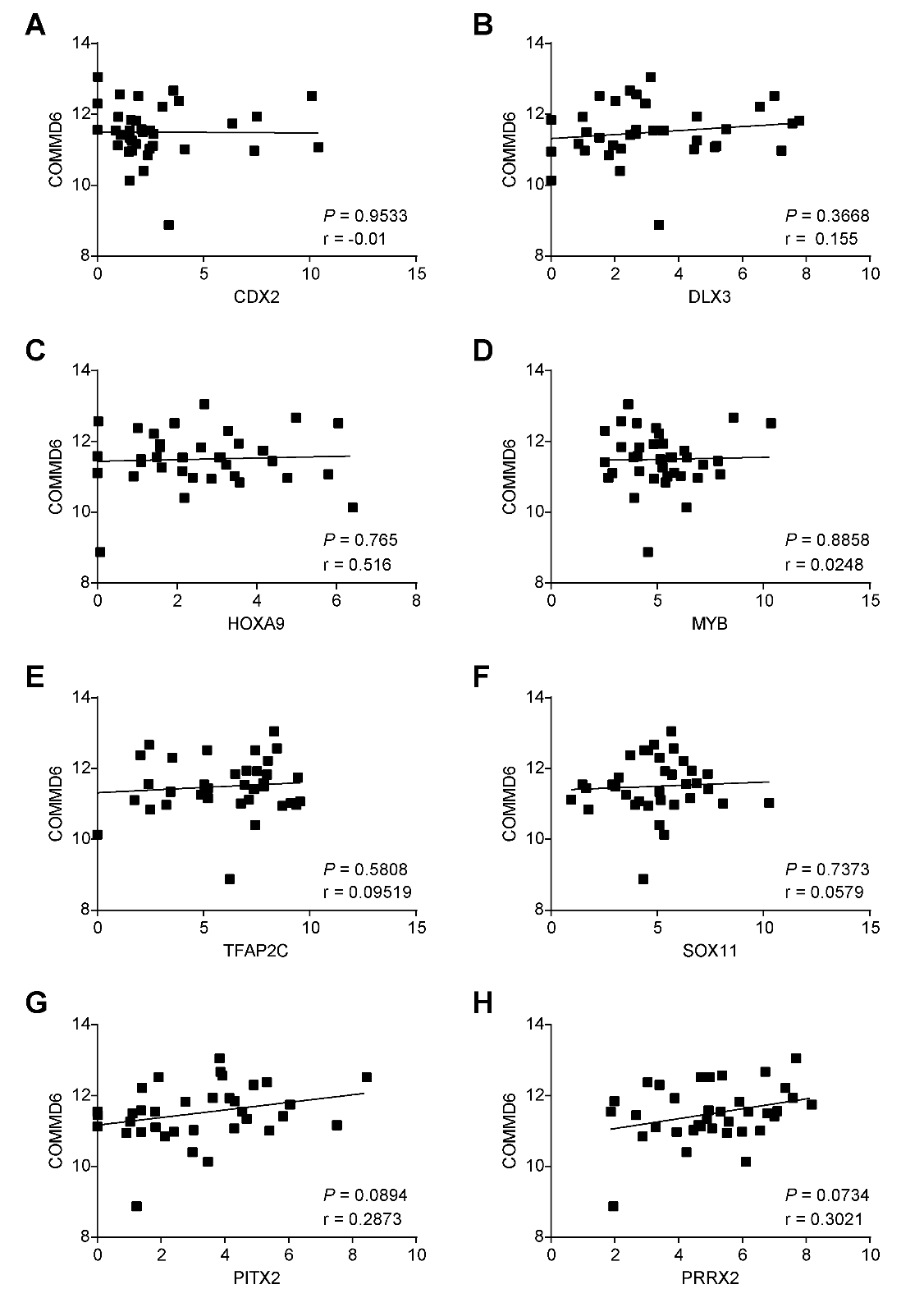
**

**Figure S7 Spearman analysis of the correlation between COMMD6 and predicted transcription factors. (A-H)** Pearson analysis of the correlation between the expression of COMMD6 and the predicted transcription factors, including CDX2, DLX3, HOXA9, MYB, TFAP2C, SOX11, PITX2 and PRRX2 in CHOL derived from TCGA database.

**
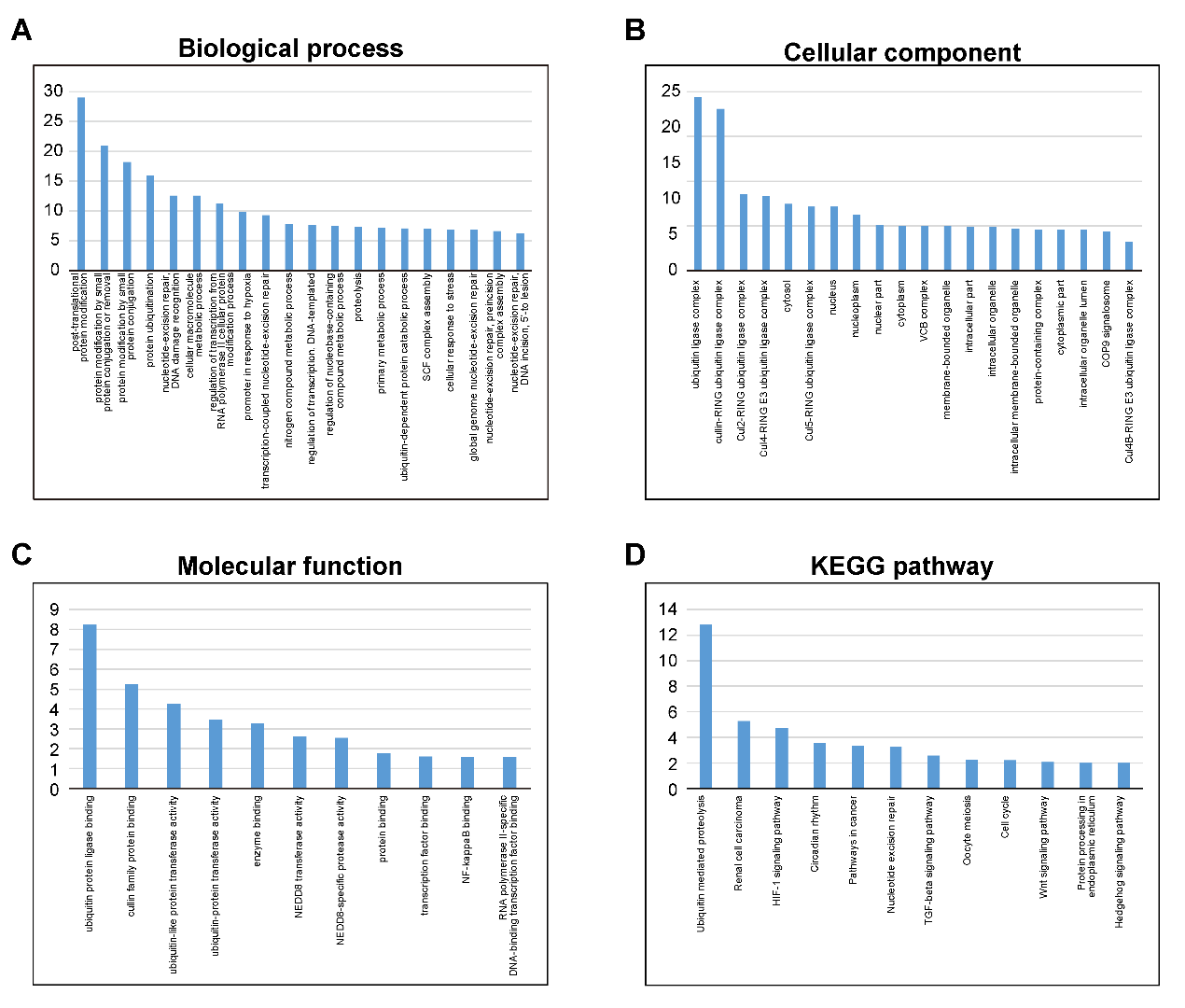
**

**Figure S8 GO and KEGG pathway enrichment analysis of COMMD6. (A-C)** Annotation of the biological function of COMMD6 interacting genes using DAVID online tools at three levels of Gene Ontology: (A) biological process, (B) cellular component and (C) molecular function. **(D)** KEGG pathway enrichment analysis of COMMD6 interacting genes.


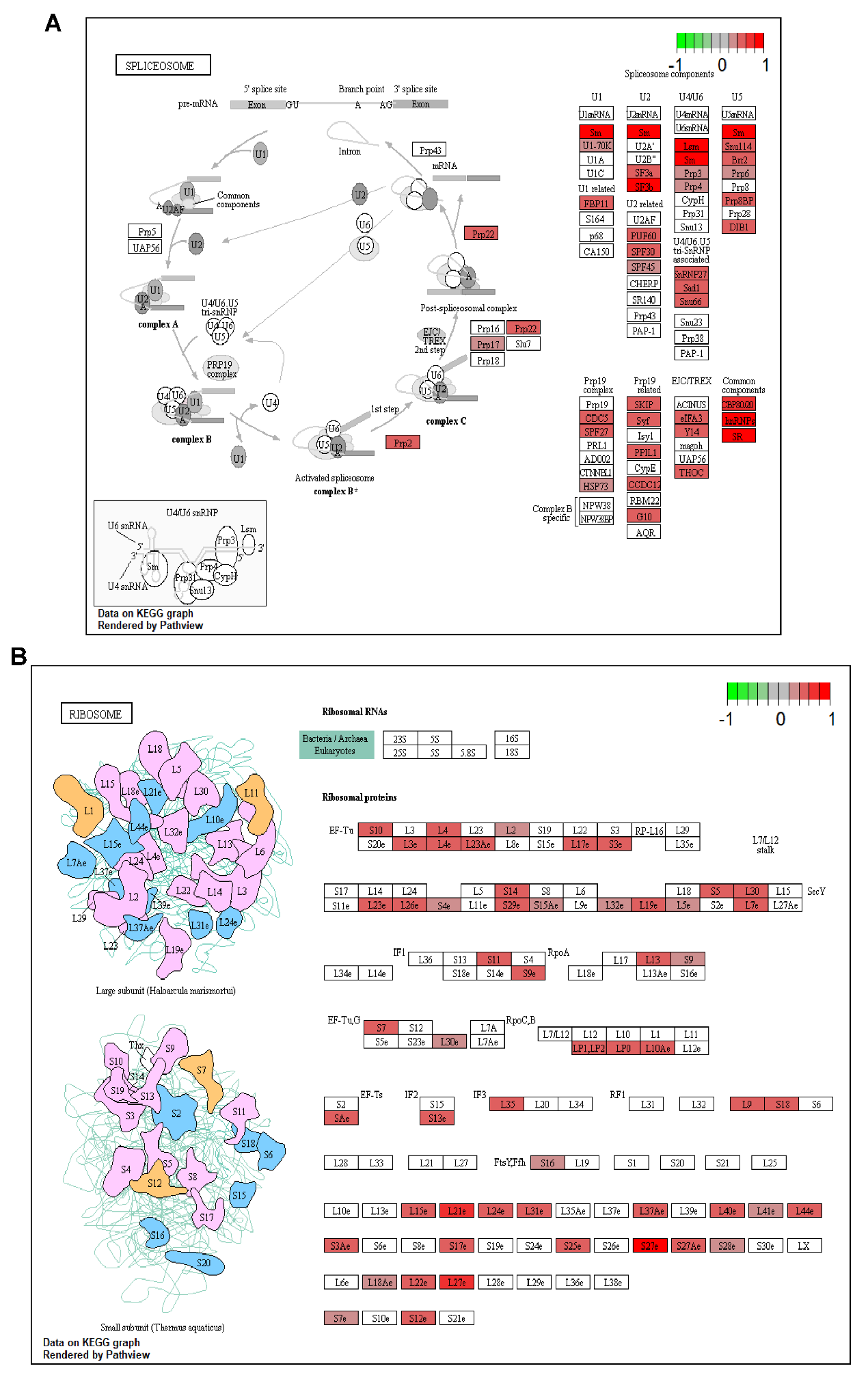


**Figure S9 KEGG analysis of COMMD6 associated signaling pathways. (A&B)** KEGG pathway enrichment analysis of COMMD6 associated spliceosome (A) and ribosome (B) signaling pathways.
